# Echocardiographic findings associated with mortality or transplant in patients with pulmonary arterial hypertension: A systematic review and meta-analysis

**DOI:** 10.1007/s12471-016-0845-3

**Published:** 2016-05-17

**Authors:** V.J.M. Baggen, M.M.P. Driessen, M.C. Post, A.P. van Dijk, J.W. Roos-Hesselink, A.E. van den Bosch, J.J.M. Takkenberg, G.T Sieswerda

**Affiliations:** 1 Department of Cardiology, University Medical Centre Utrecht, PO Box 85500, 3508 GA Utrecht, The Netherlands; 2 Department of Cardiology, Erasmus Medical Centre, Rotterdam, The Netherlands; 3 Department of Cardiology, St. Antonius Hospital, Nieuwegein, The Netherlands; 4 Department of Cardiology, Radboud University Medical Centre, Nijmegen, The Netherlands; 5 Department of Cardio-Thoracic Surgery, Erasmus Medical Centre, Rotterdam, The Netherlands

**Keywords:** Pulmonary arterial hypertension, Prognosis, Echocardiography, Mortality, Non-invasive imaging, CRD42014009231.

## Abstract

**Background:**

Identification of patients at risk of deterioration is essential to guide clinical management in pulmonary arterial hypertension (PAH). This study aims to provide a comprehensive overview of well-investigated echocardiographic findings that are associated with clinical deterioration in PAH.

**Methods:**

MEDLINE and EMBASE databases were systematically searched for longitudinal studies published by April 2015 that reported associations between echocardiographic findings and mortality, transplant or clinical worsening. Meta-analysis using random effect models was performed for echocardiographic findings investigated by four or more studies. In case of statistical heterogeneity a sensitivity analysis was conducted.

**Results:**

Thirty-seven papers investigating 51 echocardiographic findings were included. Meta-analysis of univariable hazard ratios (HRs) and sensitivity analysis showed that presence of pericardial effusion (pooled HR 1.70; 95 % CI 1.44–1.99), right atrial area (pooled HR 1.71; 95 % CI 1.38–2.13) and tricuspid annular plane systolic excursion (TAPSE; pooled HR 1.72; 95 % CI 1.34–2.20) were the most well-investigated and robust predictors of mortality or transplant.

**Conclusions:**

This meta-analysis substantiates the clinical yield of specific echocardiographic findings in the prognostication of PAH patients in day-to-day practice. In particular, pericardial effusion, right atrial area and TAPSE are of prognostic value.

**Electronic supplementary material:**

The online version of this article (doi: 10.1007/s12471-016-0845-3) contains supplementary material, which is available to authorized users.

## Introduction

The ongoing research on pulmonary arterial hypertension (PAH) has led to increased awareness of the pathophysiological, haemodynamic and clinical consequences of this devastating disease [1]. Without intervention, progressive remodelling of the distal pulmonary arteries leads to elevated pulmonary vascular resistance, eventually resulting in right heart failure and death [1, 2]. Fortunately, advances in therapeutic modalities have greatly improved the survival and quality of life in patients with PAH [3]. However, the natural course of the disease varies widely between individuals, as some patients live for decades while others die within months of diagnosis [4]. In order to guide optimal clinical management, it is therefore essential to accurately monitor disease progression and estimate prognosis in PAH.

Previously reported predictors of mortality include aetiology of PAH, gender and several functional, haemodynamic and biochemical variables [5–8]. Echocardiography is the most readily available cardiac imaging modality and is universally used in the follow-up of patients with PAH. Current literature reports several echocardiographic findings that may provide important prognostic information. The goal of this study is to provide a comprehensive overview of the most thoroughly investigated baseline echocardiographic findings that are associated with adverse clinical outcome in PAH. Separately, this study evaluates the prognostic value of a *change* in echocardiographic findings during a follow-up period.

## Methods

This systematic review was conducted in accordance with the PRISMA statement [9]. A pre-defined review protocol, as adopted by this study, can be accessed through PROSPERO (registration number: CRD42014009231).

### Literature search strategy

A comprehensive systematic search was performed in MEDLINE (via PubMed interface) and EMBASE electronic databases on 29 April 2015 using combinations of all synonyms for: PAH, echocardiography and relevant clinical outcomes (components of the Dana Point Time To Clinical Worsening composite endpoint) [1]. A validated prognostic search filter with the highest sensitivity (98 %) was added to the search syntax [10]. No language or publication period restrictions were applied. The full original search syntax is provided in Supplementary File 1.

### Selection of papers

**Fig. 1:**
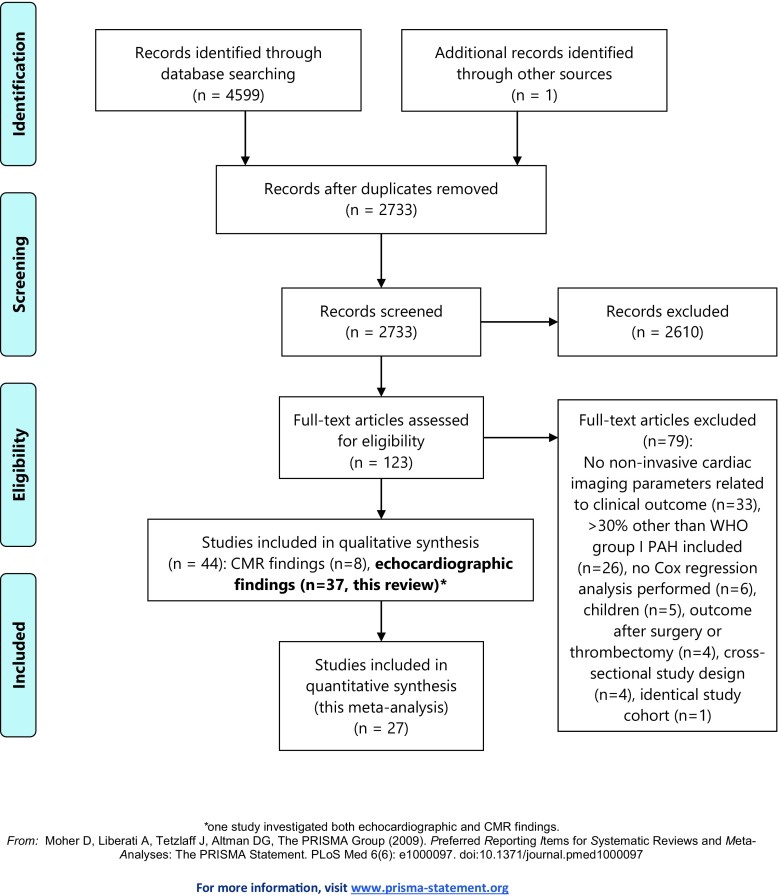
PRISMA 2009 flow diagram

A flow diagram of the selection process is shown in Fig. 1 [9]. After deduplication, one author performed screening and selection of articles based on title and abstract, using the following exclusion criteria: inappropriate study type (cross-sectional or trial design, reviews, case reports with < 10 patients, editorials or congress abstracts), non-clinical data (technical, animal and in-vitro studies), study population without PAH (e. g. acute pulmonary embolism, exercise-induced pulmonary hypertension), studies that included children < 12 years, and studies that did not relate echocardiographic findings to clinical outcome. Full-text screening was performed by two authors; reasons for exclusion are described in Fig. 1. All references of the excluded reviews and included articles were cross-checked to identify possible relevant articles missed in the original search syntax.

### Assessment of methodological quality

Study quality was critically appraised using previously developed criteria for prognostic studies [11]. We assessed study design, missing data and loss to follow-up (selection bias), adequate description and measurement of imaging features and outcome (information bias), reported effect size, treatment of continuous risk predictors and multivariable adjustment for possible confounders.

### Data extraction and analysis

Study characteristics and hazard ratios (HRs) for all investigated echocardiographic findings with accompanying 95 % confidence intervals were extracted using a standardised form. Meta-analysis was performed for all echocardiographic findings that were investigated as continuous parameters by four or more studies, using random effect models. In order to unify the extracted data to allow more studies to be pooled, HRs were recalculated to one uniform clinically applicable number of units change. Heterogeneity was assessed using Cochran’s Q test and the I^2^ statistic. Imaging findings investigated as dichotomous variables were additionally presented at the bottom of the corresponding forest plots. For all echocardiographic measurements with significant heterogeneity (I^2^ > 50 % or Cochran’s Q *p*‑value < 0.10) a sensitivity analysis was performed by excluding specific patient subgroups.

If study data were used in multiple papers and the same echocardiographic findings were evaluated, only the study with the largest sample size was used to exclude the risk of using duplicate data in our meta-analysis. The risk of publication bias was assessed using visual inspection of funnel plots and Egger’s test.

## Results

### Search results

The systematic literature search in MEDLINE and EMBASE and extensive reference cross-checking retrieved 2,733 potentially relevant records, of which 2,610 were excluded based on title and abstract (Fig. 1). After full-text review of the remaining 123 articles, 37 papers were finally selected [6, 12–47]. Study and patient characteristics of the included studies are shown in Tab. 1.

**Tab. 1:** Study characteristics

Study, ref no	Size, *n*	Age, years	Gender, % female	NYHA class III–IV, %	IPAH/hereditary	Drug/toxin	PAH-CTD	Po-PAH	PAH-CHD	WHO I PAH (other/not specified)	WHO III (lung disease)	WHO IV (CTEPH)	Follow-up duration, months	Events, *n* (%)
[11]	26	41 [16–70]	69	NR	100	-	–	–	–	–	–	–	24 ± 14	16 (62)
[12]	26	43 ± 17	73	58	100	–	–	–	–	–	–	–	NR	6 (23)
[13]	53	45 ± 14	72	70	100	–	–	–	–	–	–	–	35 [NR]	32 (60)
[14]	43	37 [14–67]	70	86	100	–	–	–	–	–	–	–	21 ± 16	12 (28)
[15]	25	38 ± 13	76	100	100	–	–	–	–	–	–	–	12 [0–84]	13 (52)
[16]	81	40 ± 15	73	100	100	–	–	–	–	–	–	–	36 ± 15	41 (51)
[17]	63	55 ± 15	83	70	37	–	38	–	–	–	21	5	19 [10–22]^a^	23 (37)
[18]	54	52 ± 11	76	76	100	–	–	–	–	–	–	–	50 [NR]	12 (22)
[19]	50	46 ± 13	78	42	46	–	22	–	–	4	–	28	14 [12–18]	19 (38)
[6]	2716	50 ± 17	79	54	49	5	24	5	12	5	–	–	17 [0–24]	340 (13)
[20]	76	61 ± 11	84	53	–	–	100	–	–	–	–	–	36 [NR–113]	42 (55)
[21]	32	53 ± 16	66	91	69	16	6	–	9	–	–	–	21 [NR]	17 (53)
[22]	59	46 ± 16	63	66	100	–	–	–	–	–	–	–	52 [28–79]^a^	23 (39)
[23]	72	52 ± 16	72	76	100	–	–	–	–	–	–	–	38 [14–71]^a^	22 (31)
[24]	484	52 ± 15	75	71	56	–	24	11	9	–	–	–	38 [16–60]	264 (55)
[25]	50	61 ± 11	98	70	–	–	100	–	–	–	–	–	16 [9–39]	25 (50)
[26]	80	56 ± 14	76	72	43	–	41	10	–	6	–	–	24 [NR]	33 (41)
[27]	95	31 ± 10	64	56	100	–	–	–	–	–	–	–	21 ± 15	27 (28)
[28]	57	52 ± 14	28	100	63	–	18	11	–	5	–	3	25 ± 29	29 (51)
[29]	181	39 ± 13	67	67	–	–	–	–	100	–	–	–	16 [7–46]	19 (10)
[30]	154	54 ± 9	84	NR	46	1	40	5	6	3	–	–	36 [17–71]^a^	71 (46)
[31]	61	48 ± 18	84	69	100	–	–	–	–	–	–	–	NR	NR
[32]	142	59 ± 15	65	44	31	9	19	4	9	1	–	27	11 [6–39]	28 (20)
[33]	577	53 ± 15	75	70	–	–	–	–	–	100	–	–	57 ± 50	NR
[34]	406	59 ± 16	65	46	–	–	–	–	–	74	14	12	16 [8–20]^a^	73 (18)
[35]	32	39 ± 15	69	59	22	–	16	–	53	–	–	9	14 [8–21]	15 (47)
[36]	124	54 ± 16	70	92	–	–	–	–	–	84	–	16	36 ± 22	31 (25)
[37]	71	57 ± 14	76	75	46	–	41	6	–	7	–	–	24 [NR]	20 (28)
[38]	50	56 ± 12	84	72	42	–	38	14	–	6	–	–	48 [NR]	NR
[39]	102	54 ± 16	84	NR	47	–	24	–	–	29	–	–	44 [22–79]^a^	43 (42)
[40]	37	46 ± 14	76	35	65	–	5	–	24	5	–	–	16 [13–18]^a^	7 (19)
[41]	48	44 ± 14	83	100	67	–	21	6	6	–	–	–	53 [21–80]^a^	18 (38)
[42]	91	42 ± 14	60	73	–	–	–	–	100	–	–	–	46 [4–64]	24 (26)
[43]	79	48 [24–65]	66	92	92	–	8	–	–	–	–	–	NR [12–92]	27 (34)
[44]	121	60 ± 14	66	63	39	–	36	18	–	6	–	–	37 ± 36	49 (40)
[45]	200	54 ± 15	71	50	47	1	33	7	12	–	–	–	43 ± 31	106 (53)
[46]	51	60 ± 15	73	71	33	–	55	6	6	–	–	–	36 ± 24	8 (16)

*CHD* congenital heart disease, *CTD* connective tissue disease, *CTEPH* chronic thromboembolic pulmonary hypertension, *HF* heart failure, *IPAH* idiopathic pulmonary arterial hypertension, *NYHA* New York Heart Association, *PAH* pulmonary arterial hypertension, *PH* pulmonary hypertension, *SD* standard deviation, *NR* not reported, *WHO* World Health Organization. ^a^interquartile range, otherwise reported as median [range] or mean ± SD

The total number of patients per study ranged from 25 to 2,716, with a mean age ranging from 31–61 years (60–98 % female) and 35–100 % of patients in New York Heart Association (NYHA) class III–IV. Twelve studies included patients with congenital heart disease (CHD) (6–100 % of patients) [6, 22, 25, 30, 31, 33, 36, 41–43, 46, 47] and seven studies included a subset of patients with pulmonary hypertension group III or IV (< 30 % of total study population) [18, 20, 29, 33, 35–37]. The majority of studies used death or transplant as primary outcome; only five studies (14 %) used a composite outcome, additionally including hospitalisation for heart failure [20, 35, 41, 47], need for a second vasodilator drug or worsening of functional class [36]. Mean follow-up duration varied between 11 and 53 months, with the primary outcome event occurring in 6–340 patients (10–62 % of study population).

### Methodological aspects

**Fig. 2:**
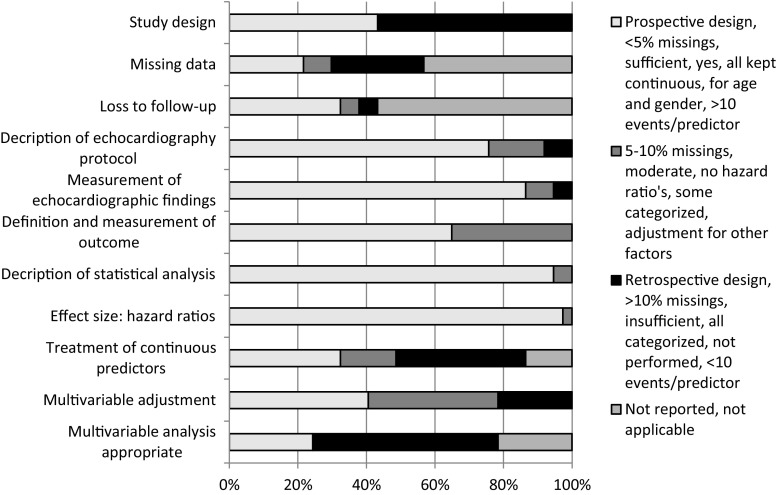
Methodological quality of the included studies. Methodological quality of the included studies was assessed on the following domains of potential bias: completeness of data (selection bias), standardisation of prognostic factors and study outcome (information bias) and statistical calculation of effect size (study outcome)

In Fig. 2, an overview of the methodological quality of all included studies is presented. Individual bias assessment per study is provided in Supplementary File 2. Studies prospectively included consecutive patients diagnosed with the disease (43 % of studies) or retrospectively reviewed echocardiographic images. Information on missing values and loss to follow-up was not reported in 16 (43 %) and 21 (57 %) of the 37 studies, respectively. It is therefore important to recognise the possible impact of selection bias on individual study outcomes.

All studies used right heart catheterisation for the diagnosis of PAH in 100 % of the included patients, except for one study that used right heart catheterisation in 87 % and echocardiography in 13 % of patients [35]. Definition and measurement of echocardiographic findings and study outcome was appropriate and consistent in the majority of studies, therefore the impact of information bias is assumed to be small.

Cox regression analysis was performed in all studies; however large differences for predictors included in the multivariable analysis were found. Twenty-nine studies performed some form of multivariable adjustment, of which only 15 adjusted for age and gender. Only nine studies (24 %) used more than ten events per predictor. Because of this large variety between studies and overall poor methodological quality of multivariable adjustment, it was chosen to present only the univariable HRs in forest plots.

### Prognostic value of baseline echocardiographic findings

**Fig. 3:**
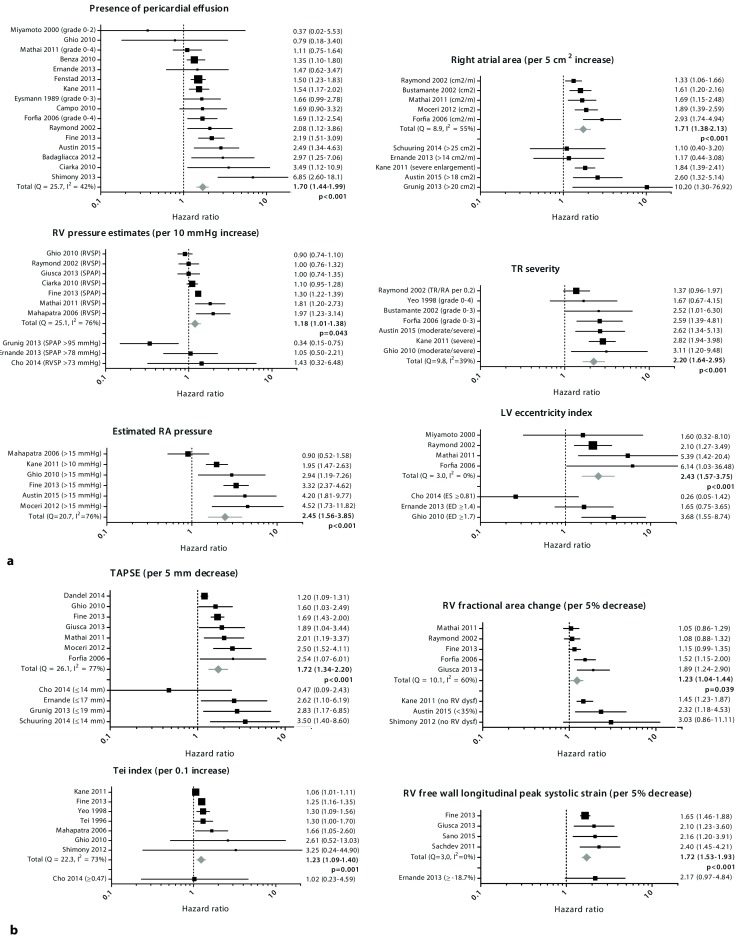
**a** Prognostic value of echocardiographic findings investigated in four or more studies. **b** Prognostic value of echocardiographic measurements of RV function investigated in four or more studies

In 37 studies, in total 51 echocardiographic findings were evaluated (Supplementary File 3). Meta-analysis was performed for ten echocardiographic findings that were suitable for pooling of results among four or more studies: presence of pericardial effusion, right atrial area, right ventricular (RV) pressure estimates, severity of tricuspid regurgitation, estimated right atrial pressure, left ventricular (LV) eccentricity index (Fig. 3a), tricuspid annular plane systolic excursion (TAPSE), RV fractional area change, Tei index (an index of RV myocardial performance) and RV free wall longitudinal peak systolic strain (LPSS) (Fig. 3b).

**Fig. 4:**
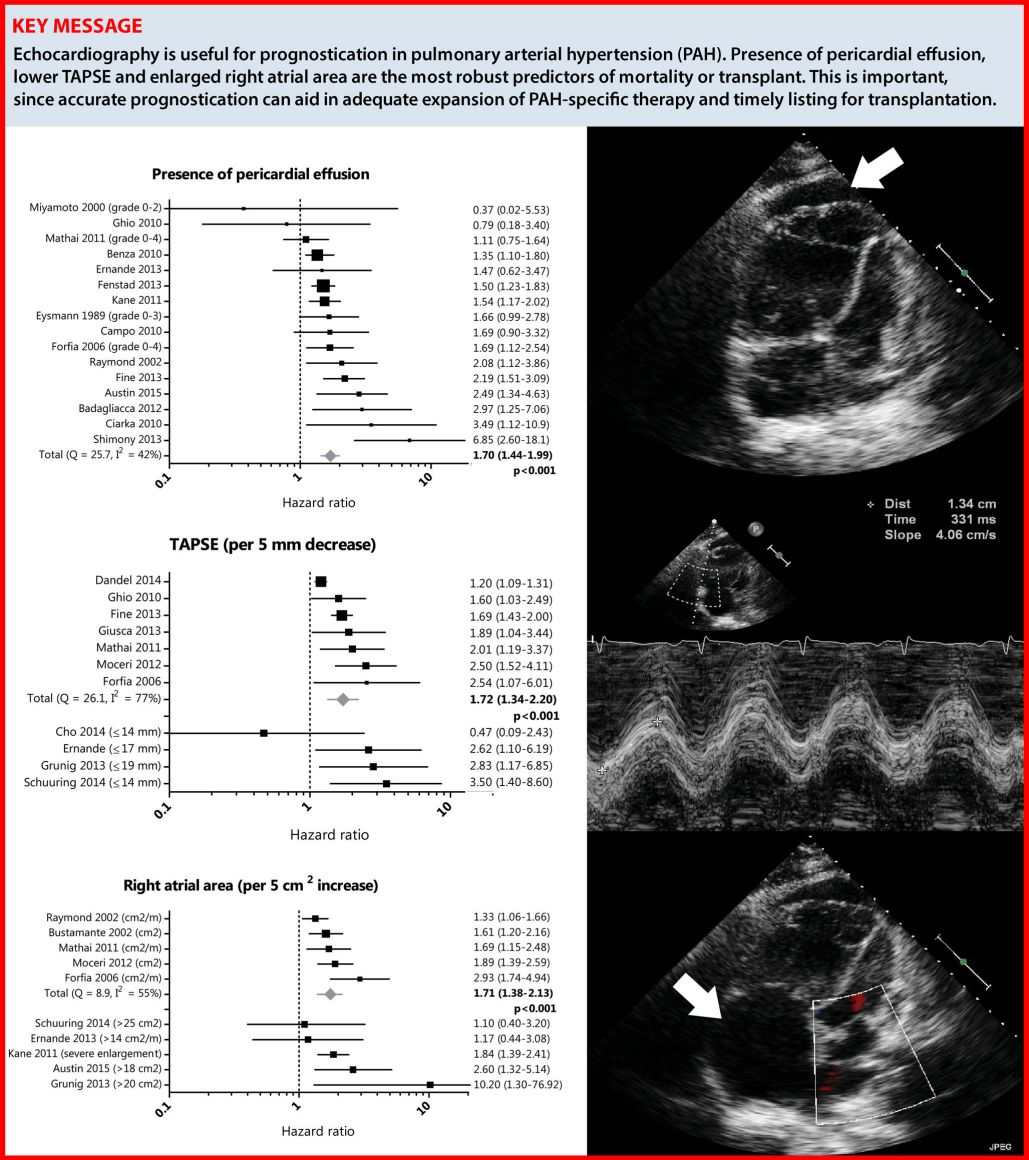
Prognostic value of pericardial effusion, TAPSE and right atrial area

Although not included in the meta-analysis, RV end-diastolic basal dimension [24, 30, 33, 36] or area [17, 25, 36, 37] and tissue Doppler velocity (S’) of the tricuspid valve annulus [30, 33, 35, 36, 41] were investigated by several studies and could be of prognostic importance. Less investigated echocardiographic measurements such as pulmonary artery capacitance [19, 35], several strain values [27, 38, 44], RV diastolic dysfunction [12, 20, 30], LV end-diastolic volume [23], systolic pulmonary artery pressure increase during exercise [37] and RV load adaptation index [44] seem promising but require further evaluation.

### Serial echocardiographic evaluation

Five studies included in this review investigated the prognostic value of a *change* in echocardiographic findings during a follow-up period, rather than their absolute baseline values, as indicated in Supplementary File 3 [39, 42, 44, 46, 47]. Patients with ≥ 5 % improvement in RV free wall LPSS on PAH treatment at 6 ± 2 months follow-up had a significantly reduced mortality risk at four years (HR 0.13; 95 % CI 0.03–0.50) [39]. Tonelli et al. showed that overall mortality was associated with a 10 % increase in RV end-diastolic area (HR 1.37; 95 % CI 1.08–1.75), tricuspid regurgitation velocity (HR 1.72; 95 % CI 1.12–2.70) and difference in qualitative RV function (HR per unit of improvement 0.55; 95 % CI 0.31–0.96) at one-year follow-up [42]. Sano et al. recently reported that a mid-term change in RV end-systolic area (HR 0.92; 95 % CI 0.86–0.98) and in right atrial area (HR 0.95; 95 % CI 0.92–0.99) were significantly related to long-term outcome [47]. In addition, changes in RV load-adaptation index and pericardial effusion have been associated with clinical outcomes in PAH [44, 46].

### Risk of bias assessment

Heterogeneity statistics (Cochran’s Q and I^2^) are presented in the corresponding forest plots in Fig. 3a and b. For all echocardiographic measurements with significant statistical heterogeneity, we performed a sensitivity analysis to evaluate possible sources for heterogeneity (Tab. 2). We excluded specific studies (< 70 % in NYHA class III–IV, < 100 % PAH, inclusion of CHD, < 50 % on PAH medication at baseline, other endpoints than mortality or transplant used) to investigate whether this impacted the pooled HR. For presence of pericardial effusion, right atrial area and TAPSE, sensitivity analysis did not change the overall conclusions. These results can therefore be regarded with a higher degree of certainty (Fig. 4).

**Tab. 2:** Sensitivity analysis for all echocardiographic measurements with significant statistical heterogeneity (I^2^ > 50 % or *p*‑value < 0.10) in specific study subgroups

	No. of studies	HR	95 % CI	*p*-value	I2, %	Cochran’s Q (*p*-value)
**Presence of pericardial effusion**	**16**	**1.70**	**1.44–1.99**	**< 0.001**	**42**	**25.7 (0.041)**
> 70 % NYHA class III–IV	8	1.56	1.31–1.86	< 0.001	23	9.0 (0.249)
100 % PAH	12	1.62	1.34–1.96	< 0.001	45	20.1 (0.045)
Exclusion of CHD	12	1.81	1.45–2.25	< 0.001	49	21.5 (0.028)
> 50 % on PAH medication/NR	10	1.86	1.40–2.47	< 0.001	60	22.3 (0.008)
Mortality/transplant as outcome	15	1.64	1.39–1.94	< 0.001	38	22.5 (0.069)
**Right atrial area, per 5 cm** ^2^ ** increase**	**5**	**1.71**	**1.38–2.13**	**< 0.001**	**55**	**8.9 (0.063)**
> 70 % NYHA class III–IV	4	1.69	1.29–2.21	< 0.001	61	7.8 (0.051)
100 % PAH	4	1.56	1.33–1.84	< 0.001	17	3.6 (0.306)
Exclusion of CHD	4	1.69	1.29–2.21	< 0.001	61	7.8 (0.051)
> 50 % on PAH medication/NR	3	1.77	1.17–2.68	0.007	74	7.7 (0.021)
Mortality/transplant as outcome	5	1.71	1.38–2.13	< 0.001	55	8.9 (0.063)
**RV pressure, per 10 mmHg increase**	**7**	**1.18**	**1.01–1.38**	**0.043**	**76**	**25.1 (< 0.001)**
> 70 % NYHA class III–IV	4	1.33	1.00–1.77	NS	72	10.9 (0.012)
100 % PAH	5	1.20	0.95–1.52	NS	75	15.8 (0.003)
Exclusion of CHD	5	1.26	1.00–1.59	NS	81	20.6 (< 0.001)
> 50 % on PAH medication/NR	5	1.19	1.02–1.38	0.024	66	11.8 (0.019)
Mortality/transplant as outcome	5	1.20	0.95–1.52	NS	75	15.8 (0.003)
**Right atrial pressure, > 15 mmHg**	**6**	**2.45**	**1.56–3.85**	**< 0.001**	**76**	**20.7 (< 0.001)**
> 70 % NYHA class III–IV	2	1.38	0.65–2.92	NS	82	5.7 (0.017)
100 % PAH	6	2.45	1.56–3.85	< 0.001	76	20.7 (< 0.001)
Exclusion of CHD	4	2.41	1.16–4.98	0.018	82	17.0 (< 0.001)
> 50 % on PAH medication/NR	0	–	–	–	–	–
Mortality/transplant as outcome	5	2.28	1.33–3.92	0.003	72	14.2 (0.007)
**TAPSE, per 5 mm decrease**	**7**	**1.72**	**1.34–2.20**	**< 0.001**	**77**	**26.1 (< 0.001)**
> 70 % NYHA class III–IV	3	1.63	1.01–2.63	0.047	69	6.5 (0.039)
100 % PAH	4	1.67	1.15–2.44	0.007	77	12.8 (0.005)
Exclusion of CHD	5	1.58	1.22–2.06	< 0.001	79	18.7 (< 0.001)
> 50 % on PAH medication/NR	4	3.24	1.92–5.45	< 0.001	0	1.2 (0.756)
Mortality/transplant as outcome	5	1.76	1.22–2.52	0.002	74	15.4 (0.004)
**RV FAC, per 5 % decrease**	**5**	**1.23**	**1.04–1.44**	**0.039**	**60**	**10.1 (0.039)**
> 70 % NYHA class III–IV	3	1.18	0.96–1.44	NS	60	5.0 (0.080)
100 % PAH	2	1.06	1.04–1.09	< 0.001	0	0.0 (0.863)
Exclusion of CHD	4	1.16	1.02–1.32	0.026	41	5.0 (0.168)
> 50 % on PAH medication/NR	5	1.23	1.04–1.44	0.039	60	10.1 (0.039)
Mortality/transplant as outcome	3	1.18	0.96–1.44	NS	60	5.0 (0.080)
**Tei index, per 0.1 unit increase**	**7**	**1.23**	**1.09–1.40**	**0.001**	**73**	**22.3 (0.001)**
> 70 % NYHA class III–IV	3	1.22	0.99–1.51	NS	76	8.3 (0.016)
100 % PAH	6	1.25	1.05–1.48	0.012	58	12.0 (0.035)
Exclusion of CHD	5	1.46	1.24–1.72	< 0.001	0	2.3 (0.677)
> 50 % on PAH medication/NR	2	1.25	1.16–1.35	< 0.001	0	0.5 (0.775)
Mortality/transplant as outcome	6	1.25	1.05–1.48	0.012	58	12.0 (0.035)

*CHD* congenital heart disease, *CI* confidence interval, *FAC* fractional area change, *HR* hazard ratio, *NR* not reported, *NS* non-significant, *NYHA* New York Heart Association, *PAH* pulmonary arterial hypertension, *RV* right ventricular, *TAPSE* tricuspid annular plane systolic excursion

No sensitivity analysis was performed for severity of tricuspid regurgitation, LV eccentricity index and RV free wall LPSS; however the forest plots show that especially tricuspid regurgitation severity and LV eccentricity index have relatively large standard errors, and thus provide imprecise risk estimations.

A combination of visual assessment of funnel plots and Egger’s test provided statistical evidence of publication bias for TAPSE (*p* = 0.026), right atrial area (*p* = 0.027) and the Tei index (*p* = 0.076 and based on the funnel plot). This may indicate that studies with a positive result are overrepresented, subsequently leading to a relative overestimation of the pooled HR in the meta-analysis.

## Discussion

To our knowledge, this is the first systematic review and meta-analysis on the prognostic value of specific echocardiographic findings in patients with PAH. Among 51 echocardiographic findings investigated in 37 studies, meta-analysis and additional sensitivity analysis showed that presence of pericardial effusion, right atrial area and TAPSE were the most robust predictors of mortality or transplant in patients with PAH.

### Right ventricular decompensation

Most deaths in patients with PAH are due to right heart failure [7]. Once the right ventricle starts to fail, it is no longer able to overcome the high pulmonary arterial pressures. This will cause a progressive rise in RV diastolic pressure and right atrial pressure, generally accompanied by right atrial enlargement. It is thought that elevated right atrial pressure causes impaired lymphatic and venous drainage, subsequently leading to pericardial fluid accumulation [17]. These insights into the mechanistic course tilting a stable PAH state towards death clearly explain why pericardial effusion, right atrial area, estimated right atrial pressure and RV dysfunction measured on echocardiography are associated with mortality in PAH.

Studies investigating RV function and right atrial pressure as assessed with other diagnostic modalities, such as cardiac magnetic resonance imaging and right heart catheterisation, report comparable findings [6, 7, 48, 49]. However, as echocardiography is more widely applied, non-invasive and less expensive, it is more suitable for the evaluation of PAH patients in day-to-day practice.

### Serial measurements

The majority of the included studies investigated the prognostic value of baseline imaging findings, evaluated at the time of diagnosis. Complementary information on *changes* in haemodynamic, functional and biochemical variables may better reflect an individual’s response to PAH-targeted therapy – or progression of disease [50]. Interestingly, although the first study included in this review originates from 1989, serial echocardiographic evaluation in PAH has only recently gained scientific attention, as Hardegree and colleagues were the first ones to publish on this topic in 2013 [12, 39]. Thus far, changes in pericardial fluid accumulation, right atrial area, tricuspid regurgitation velocity, RV free wall LPSS and qualitative RV function, RV dimensions and RV load-adaptation index have been associated with clinical outcomes in PAH [39, 42, 44, 46, 47]. This is in line with the conclusions reached by the international working group of Vonk-Noordegraaf et al., who stated that changes in RV imaging parameters after treatment reflect altered exercise capacity and predict subsequent survival [51]. Advantages of echocardiography over more expensive or invasive imaging modalities become especially important in the serial evaluation of individual patients.

### Heterogeneity

PAH prognosis depends largely on the underlying aetiology, as the right ventricle can show rapid deterioration after initial diagnosis in patients with idiopathic or connective tissue disease-PAH, while it may cope successfully with pressure overload for decades in patients with congenital heart disease [4, 6]. Still, most studies in this review investigated the World Health Organisation (WHO) group I PAH as a whole. Moreover, some studies in this review *additionally* included small subsets (< 30 %) of patients with WHO group III (pulmonary hypertension due to lung disease) or IV (chronic thromboembolic pulmonary hypertension), which further increases the heterogeneity of the study population. The heterogeneity of pulmonary hypertension aetiologies among studies in this review likely plays a major role in the observed variation between the reported study results. Along the same line, disease severity and changing available treatment options over time likely contribute to this observed heterogeneity across studies. We therefore conducted a sensitivity analysis in which we excluded studies with other than WHO group I PAH, patients with CHD, < 70 % patients in NYHA class III–IV (thus investigating a sicker patient population) or < 50 % on PAH medication (representing other available treatment options). Importantly, this clearly reduced the statistical heterogeneity in specific study subgroups; however the overall conclusions for presence of pericardial effusion, right atrial area and TAPSE remained unchanged.

### Study limitations

We presented only univariable HRs in this study, because of the large variety between studies in which multivariable adjustment was performed (regarding the type and number of predictors per event used). Second, formal tests for publication bias retrieved significant results for the variables right atrial area, TAPSE and Tei index. Theoretically, publication bias may cause underreporting of non-significant HRs, leading to a relative overestimation of the pooled HRs. Exact results of the random effect models as presented in this review should be therefore interpreted with caution.

### Clinical implications

In order to adequately expand PAH-specific therapy and timely list patients for transplantation, accurate prognostication is highly important. The data in this review imply that especially pericardial effusion, enlarged right atrial area and decreased TAPSE are useful echocardiographic markers to predict mortality or transplantation. This is largely concordant with the 2015 European Society of Cardiology/European Respiratory Society Guidelines for the diagnosis and treatment of pulmonary hypertension, in which right atrial area and pericardial effusion are recommended as determinants of prognosis [2]. Controversy continues to exist about the use of TAPSE; it has been suggested that progressive RV dysfunction is associated with a decline in TAPSE until a certain floor effect is reached [52]. Of note, considering the multi-faceted nature of this disease, accurate prognostication should always be based on a combination of haemodynamic, functional, biochemical and echocardiographic findings, and should not rely on just one single parameter according to the current guidelines [2].

### Conclusions

This meta-analysis substantiates the clinical yield of specific echocardiographic findings in the prognostication of PAH patients in day-to-day practice. Although accurate prognostication should not rely on just one single parameter, presence of pericardial effusion, enlarged right atrial area and decreased TAPSE are the most firmly established echocardiographic tools that can be of important additional value.

## Caption Electronic Supplementary Material

Search syntax used to identify publications of interest. Search date April 29, 2015.

Risk of bias assessment in individual studies.

Overview of investigated echocardiographic findings per study.
