# Supplementary material for: Echocardiographic findings associated with mortality or transplant in patients with pulmonary arterial hypertension: A systematic review and meta-analysis
Source: Neth Heart J. 2016 May 17;24(6):374–89. doi: 10.1007/s12471-016-0845-3 (PMC4887306; doi:10.1007/s12471-016-0845-3)
Supplement: Supplementary file 1 — Search syntax used to identify publications of interest. Search date April 29, 2015. [file 12471_2016_845_MOESM1_ESM.docx]

**Echocardiographic findings associated with mortality or transplant in patients with pulmonary arterial hypertension: A systematic review and meta-analysis**

V.J.M. Baggen, M.M.P. Driessen, M.C. Post, A.P. van Dijk, J.W. Roos-Hesselink, A.E. van den Bosch, J.J.M. Takkenberg, G.Tj. Sieswerda

([G.T.Sieswerda@umcutrecht.nl](mailto:G.T.Sieswerda@umcutrecht.nl))

**Supplementary File 1.** Search syntax used to identify publications of interest. Search date April 29, 2015.

| **Population #1** | “pulmonary hypertension”[tiab] OR “pulmonary arterial hypertension”[tiab] OR “pulmonary artery hypertension”[tiab] |
| --- | --- |
| **Predictive variable #2** | echocardiograph*[tiab] OR echocardiogram[tiab] OR ultrasound[tiab] OR ultrasonograph*[tiab] OR ultrasonogram[tiab] OR ultrasonic[tiab] OR “M-Mode”[tiab] OR “M Mode”[tiab] OR doppler[tiab] OR “cardiac magnetic resonance”[tiab] OR imaging[tiab] OR MRI[tiab] OR “magnetic resonance angiography”[tiab] OR “magnetic resonance angiographies”[tiab] |
| **Outcome #3** | death[tiab] OR mortality[tiab] OR survival[tiab] OR fatal[tiab] OR fatality[tiab] OR transplant*[tiab] OR graft*[tiab] OR hospitalization[tiab] OR hospitalisation[tiab] OR admission[tiab] OR readmission[tiab] OR transcatheter[tiab] OR intervention[tiab] OR intravenous[tiab] OR infusion*[tiab] OR “new york heart association”[tiab] OR “functional class”[tiab] OR “functional status”[tiab] OR function[tiab] OR exercise[tiab] OR endurance[tiab] OR ((“6-minute”[tiab] OR “6 minute”[tiab] OR “6-min”[tiab] OR “6 min”[tiab] OR “six-minute”[tiab] OR “six minute”[tiab]) AND walk[tiab] AND (test[tiab] OR distance[tiab])) OR VO2max[tiab] OR “VO2 max”[tiab] OR VO2peak[tiab] OR “VO2 peak”[tiab] OR “aerobic capacity”[tiab] OR (oxygen[tiab] AND (uptake[tiab] OR consumption[tiab])) OR symptom*[tiab] OR morbidity[tiab] OR outcome[tiab] OR outcomes[tiab] |
| **Prognostic studies #4** | predict*[tiab] OR clinical*[tiab] OR outcome*[tiab] OR risk*[tiab] OR prognos*[tiab] |
| **Search results (#1 AND #2 AND #3 AND #4)** | MEDLINE: 2371  EMBASE: 2228 (replace [tiab] by :ti,ab) |
| **Other limits (added search strings):**  **PubMed:** NOT (animals[MeSH] NOT humans[MeSH])  **Embase:** NOT ([conference abstract]/lim OR [conference paper]/lim OR [conference review]/lim OR [editorial]/lim OR [erratum]/lim OR [letter]/lim OR [note]/lim) AND [embase]/lim | |
