# Supplementary material for: Echocardiographic findings associated with mortality or transplant in patients with pulmonary arterial hypertension: A systematic review and meta-analysis
Source: Neth Heart J. 2016 May 17;24(6):374–89. doi: 10.1007/s12471-016-0845-3 (PMC4887306; doi:10.1007/s12471-016-0845-3)
Supplement: Supplementary file 2 — Risk of bias assessment in individual studies. [file 12471_2016_845_MOESM2_ESM.docx]

**Echocardiographic findings associated with mortality or transplant in patients with pulmonary arterial hypertension: A systematic review and meta-analysis.**

V.J.M. Baggen, M.M.P. Driessen, M.C. Post, A.P. van Dijk, J.W. Roos-Hesselink, A.E. van den Bosch, J.J.M. Takkenberg, G.Tj. Sieswerda

([G.T.Sieswerda@umcutrecht.nl](mailto:G.T.Sieswerda@umcutrecht.nl))

**Supplementary File 2.** Risk of bias assessment in individual studies.

|  |  | **Selection bias** | | **Information bias: defined and measured appropriately?** | | | | **Statistical calculation of effect size** | | | |
| --- | --- | --- | --- | --- | --- | --- | --- | --- | --- | --- | --- |
| **Author, Year** | **Study design** | **Missing data** | **Loss to follow-up** | **Description of echocardiography protocol** | **Measurement of echocardiographic findings** | **Definition and measurement of outcome** | **Description of statistical analysis** | **Effect size: hazard ratios** | **Treatment of continuous predictors** | **Multivariable adjustment** | **Multivariable analysis appropriate** |
| Eysmann 1989 | + | nr | + | + | + | + | + | ± | + | - | NA |
| Tei 1996 | - | + | nr | + | + | + | + | + | - | - | NA |
| Yeo 1998 | - | - | ± | + | + | + | + | + | + | + | - |
| Miyamoto 2000 | + | nr | + | ± | ± | + | + | + | + | + | - |
| Bustamante 2002 | + | + | - | + | + | + | ± | + | ± | + | - |
| Raymond 2002 | - | - | ± | + | + | ± | + | + | - | + | - |
| Forfia 2006 | + | + | nr | + | + | + | + | + | + | ± | - |
| Mahapatra 2006 | - | - | nr | + | + | + | + | + | + | ± | - |
| Utsunomiya 2009 | - | nr | + | + | + | ± | + | + | + | - | NA |
| Benza 2010 | + | - | + | - | ± | + | + | + | NA | + | - |
| Campo 2010 | - | + | + | - | ± | + | + | + | NA | - | NA |
| Ciarka 2010 | + | nr | + | + | + | + | + | + | + | ± | - |
| Ghio 2010 | + | nr | nr | + | + | ± | + | + | ± | - | NA |
| Ghio 2011 | + | nr | nr | + | + | ± | + | + | - | ± | + |
| Kane 2011 | - | + | + | ± | - | + | + | + | - | + | + |
| Mathai 2011 | + | nr | nr | + | + | ± | + | + | + | ± | - |
| Sachdev 2011 | - | - | nr | + | + | + | + | + | - | + | + |
| Zeng 2011 | - | nr | nr | + | + | + | + | + | - | ± | - |
| Badagliacca 2012 | - | nr | nr | ± | + | + | + | + | NA | ± | - |
| Moceri 2012 | + | nr | + | + | + | + | + | + | ± | ± | - |
| Shimony 2012 | - | + | nr | - | - | ± | + | + | + | - | NA |
| Tonelli 2012 | - | nr | nr | + | + | + | + | + | ± | + | - |
| Ernande 2013 | + | nr | nr | + | + | + | + | + | - | + | - |
| Fenstad 2013 | + | + | nr | + | + | ± | + | + | NA | + | - |
| Fine 2013 | + | - | + | + | + | + | + | + | - | + | - |
| Giusca 2013 | + | - | nr | ± | + | ± | + | + | + | ± | - |
| Grunig 2013 | + | - | + | + | + | + | + | + | - | + | + |
| Hardegree 2013 | - | - | + | + | + | ± | + | + | - | ± | - |
| Hardegree 2013 | - | ± | + | + | + | ± | + | + | - | + | + |
| Shimony 2013 | - | + | nr | ± | + | ± | + | + | NA | ± | + |
| Cho 2014 | - | nr | nr | + | + | + | ± | + | - | - | NA |
| Tonelli 2014 | - | nr | nr | + | + | + | + | + | ± | + | - |
| Schuuring 2014 | + | ± | - | ± | + | + | + | + | - | ± | + |
| Dandel 2014 | - | nr | nr | + | + | ± | + | + | ± | - | NA |
| Austin 2015 | - | nr | nr | + | + | + | + | + | - | ± | + |
| Batal 2015 | - | - | nr | + | + | ± | + | + | + | + | + |
| Sano 2015 | - | ± | nr | + | + | + | + | + | + | ± | - |

**Legend**:

nr, not reported; NA, not applicable.

*Study design*: +, prospective cohort study; -, retrospective cohort study.

*Missing data*: +, <5%; ±, 5-10% or <5% selective missing data; -, >10% or >5% selective missing data.

*Loss to follow-up*: +, <5%; ±, 5-10% or <5% selective loss to follow-up; -, >10% or >5% selective loss to follow-up.

*Echocardiography protocol and statistical analysis*: +, well defined; ±, moderately defined; -, poorly defined.

*Echocardiographic findings and outcome*: +, well defined and measured appropriately; ± moderately defined or moderately measured; -, poorly defined or poorly measured.

*Effect size*: +, Cox regression model and outcomes presented as HR [95% CI]; ± Cox regression model, outcomes not presented as HR [95% CI]; -, independent-samples T-test or outcomes presented as OR/RR.

*Treatment of continuous predictors*: +, all kept continuous; ±, some categorised/dichotomised, some not; -, all categorised/dichotomised.

*Multivariable adjustment*: +, yes, at least for age and gender; ± multivariable adjustment for other factors; -, no multivariable analysis performed or not described.

*Multivariable analysis appropriate*: +, ≥10 events per predictor used; -, <10 events per predictor used.
